# Retrospective–Prospective Observational Study of Serum Magnesium Level Dynamics and Their Correction in Physiological and Complicated Pregnancies

**DOI:** 10.1155/ogi/4276911

**Published:** 2026-08-17

**Authors:** Hasmik Badalyan, Araksya Grigoryan, Georgi Okoev

**Affiliations:** ^1^ Hospital and Polyclinic No 2, “Margaryan Maternity Hospital” Research Center of Maternal and Child Health Protection, Yerevan, Armenia; ^2^ Department of Laboratory Diagnostics, Yerevan State Medical University After M. Heratsi, Yerevan, Armenia, ysmu.am; ^3^ Department of Obstetrics, Gynecology and Reproductive Medicine of Professional and Continuing Education Center, Yerevan State Medical University After M. Heratsi, Yerevan, Armenia, ysmu.am

**Keywords:** hypomagnesemia, magnesium, magnesium sulfate, micronutrients, preeclampsia, pregnancy, pregnancy complications

## Abstract

**Background and Aims:**

Magnesium is an essential micronutrient involved in numerous biochemical and physiological processes during pregnancy. The aim of this study was to evaluate serum magnesium level dynamics during physiological and complicated pregnancies and to assess the effectiveness of magnesium deficiency correction.

**Methods:**

A retrospective–prospective observational cohort study was conducted between 2020 and 2025, including 233 pregnant women. Serum magnesium levels were measured in the first and third trimesters. Participants were divided into physiological (*n* = 110) and complicated pregnancy (*n* = 123) groups. Magnesium status was classified as deficiency (< 0.72 mmol/L), insufficiency (0.72–0.80 mmol/L), and normal (> 0.80 mmol/L). Oral magnesium lactate with pyridoxine was administered in cases of deficiency. Statistical analysis was performed using SPSS software with a significance level of *p* < 0.05 (two‐sided). Effect size was assessed using Cohen’s *d*.

**Results:**

In physiological pregnancies, no statistically significant changes in serum magnesium levels were observed between trimesters (*p* > 0.05). In complicated pregnancies, a downward trend in magnesium levels was observed, reaching statistical significance in one subgroup (*p* = 0.043; *d* = 0.41). Patients with baseline deficiency or insufficiency did not achieve normalization despite therapy. Differences between treated and untreated groups were limited and inconsistent.

**Conclusion:**

Serum magnesium levels remain stable during physiological pregnancy but tend to decrease in complicated pregnancies. Standard oral magnesium supplementation demonstrates limited effectiveness in restoring normal serum levels, highlighting the need for further research and individualized therapeutic strategies.

## 1. Introduction

Magnesium is a vital micronutrient and a key cofactor for numerous biochemical processes in the human body. It is involved in more than 600 enzymatic reactions, including protein synthesis, mitochondrial function, neuromuscular transmission, bone formation, and immune system regulation [1–3].

In recent years, hypomagnesemia has been recognized as a common condition in pregnant women. According to current research, its prevalence reaches 34.0% with serum magnesium levels < 0.66 mmol/L and up to 78.9% with magnesium deficiency (< 0.8 mmol/L) [4].

Despite its high prevalence, magnesium deficiency often remains undiagnosed due to the nonspecific clinical manifestations and the lack of generally accepted, accurate diagnostic criteria [3, 5].

Low magnesium levels during pregnancy are associated with adverse maternal and perinatal outcomes [6].

In particular, its significant role in the pathogenesis of hypertensive disorders of pregnancy has been demonstrated. Mean serum magnesium levels were statistically significantly lower in pregnant women with preeclampsia compared to those with normal pregnancy (1.08 ± 0.23 versus 1.21 ± 0.32 mg/dL, *p* < 0.001) [7].

These data suggest that hypomagnesemia may be a potential risk factor for the development of preeclampsia [8, 9].

Furthermore, low magnesium levels before pregnancy and their subsequent decline during the gestational period may contribute to preterm birth [10].

A significant association has been established between hypomagnesemia and an increased incidence of preterm birth, low birth weight, and neonatal intensive care unit admissions [11]. Despite the fact that less than 1% of the total body magnesium is found in the serum, while approximately 99% is localized intracellularly, serum magnesium determination remains the most common and accessible method for assessing magnesium status in clinical practice and is considered quite informative [12].

However, the reference range for serum magnesium varies, making it difficult to interpret the results. Furthermore, there is currently no standardized and scientifically validated reference range for serum magnesium levels [13, 14].

Thus, the impact of reduced magnesium levels on pregnancy is beyond doubt; however, the issues of screening, prevention, and treatment of hypomagnesemia remain the subject of active debate. Further research is needed to evaluate the effectiveness of existing regimens for correcting magnesium deficiency in pregnant women and to substantiate the advisability of its prevention [15–18].

### 1.1. Aim of the Study

To assess the level of serum magnesium and its dynamics during physiological and complicated pregnancy and to determine the effectiveness of magnesium deficiency correction.

## 2. Materials and Methods

### 2.1. Study Design and Study Setting

A retrospective–prospective observational cohort study was conducted between 2020 and 2025 at the Maternal and Child Health Research Center (Yerevan, Armenia) and Medical Association No. 2 (Yerevan, Armenia). The study was designed to evaluate the association between serum magnesium levels measured in early pregnancy and pregnancy outcomes, as well as to analyze the dynamics of magnesium status during gestation and the effectiveness of magnesium deficiency correction therapy.

The study followed Strengthening the Reporting of Observational Studies in Epidemiology (STROBE) guidelines for observational cohort studies. All analyses were predefined, and subgroup analyses were based on clinically established pregnancy outcomes (physiological vs. complicated pregnancy).

### 2.2. Study Population

A total of 233 pregnant women aged 18–45 years with singleton pregnancies were included in the study. All participants were enrolled during the first trimester and had at least one serum magnesium measurement during pregnancy. Written informed consent was obtained from all participants prior to inclusion.

#### 2.2.1. Inclusion Criteria

• Age between 18 and 45 years.
• Singleton pregnancy.
• Enrollment in the first trimester.
• Availability of serum magnesium measurement.
• Signed informed consent.
• For treatment groups: administration of oral magnesium supplementation.
• For control groups: no magnesium therapy.

#### 2.2.2. Exclusion Criteria

• Multiple pregnancy.
• Chronic kidney disease, liver disease, or severe cardiovascular disease.
• Preexisting hypertension or diabetes mellitus before pregnancy.
• Congenital fetal anomalies.
• Use of magnesium sulfate (MgSO_4_) or other parenteral magnesium therapy during pregnancy.
• Use of drugs significantly affecting magnesium metabolism.
• Incomplete clinical or laboratory data.
• Withdrawal of consent.

### 2.3. Study Groups and Clinical Classification

Participants were divided according to pregnancy outcome:

• Physiological pregnancy: *n* = 110
• Complicated pregnancy: *n* = 123

Complicated pregnancy included cases with

• Preeclampsia
• Gestational hypertension
• Placental insufficiency
• Fetal growth restriction (FGR)
• Threatened preterm birth

### 2.4. Serum Magnesium Assessment

Serum magnesium concentrations were measured twice:

• First trimester (baseline assessment)
• Third trimester (follow‐up assessment)

Due to the absence of universally accepted pregnancy‐specific reference ranges, a clinically expanded classification was used based on published literature:

• Magnesium deficiency: < 0.72 mmol/L
• Magnesium insufficiency: 0.72–0.80 mmol/L
• Normal magnesium level: > 0.80 mmol/L

This classification reflects evidence suggesting that values below 0.75–0.80 mmol/L may indicate latent or subclinical magnesium deficiency despite being within standard laboratory reference ranges.

### 2.5. Study Group Allocation

Based on serum magnesium levels, participants were categorized as follows:

 Physiological pregnancy group (*n* = 110)
  • Group I (*n* = 11): magnesium deficiency
  • Group II (*n* = 47): magnesium insufficiency
  • Group III (*n* = 52): normal magnesium level
 Complicated pregnancy group (*n* = 123)
  • Group IV (*n* = 43): magnesium deficiency
  • Group V (*n* = 52): magnesium insufficiency
  • Group VI (*n* = 28): normal magnesium level

### 2.6. Additional Subgroup (Intrapartum Assessment)

A randomly selected subgroup of 37 women with complicated pregnancies underwent magnesium measurement during labor to identify untreated or uncorrected magnesium status:

• Group VII (*n* = 20): magnesium deficiency
• Group VIII (*n* = 12): magnesium insufficiency
• Group IX (*n* = 5): normal magnesium level

Interpretation of this subgroup was performed cautiously due to differences in timing of assessment and nonrandomized allocation.

Data on the nature and frequency of complications are presented in Table 1. The mean age of patients in the groups ranged from 25.0 ± 2.9 to 31.9 ± 6.3 years and did not differ significantly between the groups, indicating comparability in this parameter. According to the study design, groups with a physiological pregnancy course were characterized by the absence of obstetric pathology and a predominance of term deliveries, whereas in groups with a pathological pregnancy course and without magnesium therapy, a higher frequency of complications and adverse outcomes was noted.

**TABLE 1:** Clinical and obstetric characteristics of the study groups.

Group (*n*)	Therapy	Age (years), mean ± SD	Threatened miscarriage, *n* (%)	Gestational hypertension/preeclampsia, *n* (%)	FGR, *n* (%)	Threatened preterm labor, *n* (%)	Miscarriage, *n* (%)	Preterm birth, *n* (%)	Term delivery, *n* (%)
I (11)	Yes	26.9 ± 5.2	0 (0)	0 (0)	0 (0)	0 (0)	0 (0)	0 (0)	11 (100)
II (47)	Yes	28.9 ± 4.7	0 (0)	0 (0)	0 (0)	0 (0)	0 (0)	0 (0)	47 (100)
III (52)	Yes	27.1 ± 5.0	0 (0)	0 (0)	0 (0)	0 (0)	0 (0)	0 (0)	52 (100)
IV (43)	Yes	28.1 ± 6.0	17 (39.5)	3 (7.0)	0 (0)	16 (37.2)	2 (4.7)	7 (16.3)	34 (79.1)
V (52)	Yes	28.5 ± 5.8	22 (42.3)	3 (5.8)	0 (0)	22 (42.3)	2 (3.8)	8 (15.4)	42 (80.8)
VI (28)	Yes	29.0 ± 6.5	8 (28.6)	3 (10.7)	1 (3.6)	8 (28.6)	1 (3.6)	6 (21.4)	21 (75.0)
VII (20)	No	31.9 ± 6.3	11 (55.0)	5 (25.0)	3 (15.0)	4 (20.0)	0 (0)	9 (45.0)	11 (55.0)
VIII (12)	No	26.9 ± 5.5	5 (41.7)	4 (33.3)	3 (25.0)	1 (8.3)	0 (0)	3 (25.0)	9 (75.0)
IX (5)	No	25.0 ± 2.9	3 (60.0)	3 (60.0)	1 (20.0)	0 (0)	0 (0)	1 (20.0)	4 (80.0)

Abbreviations: FGR, fetal growth restriction; SD, standard deviation.

### 2.7. Magnesium Replacement Therapy

Patients diagnosed with magnesium deficiency or insufficiency received oral magnesium lactate combined with pyridoxine (vitamin B_6_) for a duration of 4 weeks.

Oral magnesium therapy was selected due to its suitability for long‐term outpatient management, gradual restoration of intracellular and serum magnesium stores, and favorable safety profile.

Although magnesium sulfate (MgSO_4_) remains the gold standard in obstetric practice for the prevention and treatment of eclampsia and for fetal neuroprotection in threatened preterm birth, it was not used in this study. Its use is associated with intravenous administration, intensive monitoring requirements, and acute clinical indications, which were not aligned with the study design focused on chronic magnesium deficiency correction.

### 2.8. Serial Monitoring

Serum magnesium levels were reassessed in the third trimester to evaluate physiological changes during pregnancy and the effect of magnesium supplementation.

### 2.9. Statistical Analysis

Statistical analysis was performed using IBM SPSS Statistics (Version 28.0). Continuous variables were expressed as mean ± standard deviation (*M* ± SD) with 95% confidence intervals (CIs). Categorical variables were expressed as frequencies and percentages.

Normality was assessed using the Shapiro–Wilk test. Depending on the distribution, an independent samples *t*‐test or Mann–Whitney *U* test was used for intergroup comparisons. A paired *t*‐test was applied for intragroup comparisons. Categorical variables were analyzed using the chi‐square test.

Effect size was calculated using Cohen’s *d* and interpreted as follows:

• Small effect: *d* < 0.2
• medium effect: *d* ≈ 0.5
• Large effect: *d* ≥ 0.8

All statistical tests were two‐tailed, and *p* < 0.05 was considered statistically significant. Missing data were handled using complete‐case analysis without imputation.

No statistically significant differences were observed in key comparative analyses. In particular:

• Group II vs. Group VIII: *p* = 0.73, Cohen’s *d* = 0.11.
• Group V vs. Group VIII: *p* = 0.35, Cohen’s *d* = −0.31.
• Group III vs. Group IX: *p* = 1.000, 95% CI overlap complete.
• Group VI vs. Group IX: *p* = 0.44, Cohen’s *d* = 0.17.

### 2.10. Ethical Considerations

The study was conducted in accordance with the Declaration of Helsinki. Ethical approval was obtained from the local institutional ethics committee. Written informed consent was obtained from all participants prior to inclusion in the study.

## 3. Results

### 3.1. Dynamics of Serum Magnesium Levels During Pregnancy

The dynamics of serum magnesium concentrations in the first and third trimesters across the study groups are presented in Table 2.

**TABLE 2:** Dynamics of serum magnesium levels in the study group (mean ± SD; 95% CI, mmol/L).

Group	*n*	I trimester (95% CI)	III trimester (95% CI)	*p*	Cohen’s *d*
I	11	0.687 ± 0.015 (0.678–0.696)	0.686 ± 0.031 (0.667–0.705)	0.927	< 0.2
II	47	0.761 ± 0.020 (0.755–0.767)	0.753 ± 0.050 (0.738–0.768)	0.24	< 0.2
III	52	0.835 ± 0.044 (0.823–0.847)	0.826 ± 0.044 (0.814–0.838)	0.16	< 0.2
IV	43	0.671 ± 0.050 (0.656–0.686)	0.681 ± 0.050 (0.666–0.696)	0.19	0.27
V	52	0.748 ± 0.054 (0.733–0.763)	0.733 ± 0.054 (0.718–0.748)	0.054	−0.29
VI	28	0.847 ± 0.083 (0.816–0.878)	0.813 ± 0.083 (0.782–0.844)	0.043	0.41

Abbreviation: CI, confidence interval.

### 3.2. Physiological Pregnancy (Groups I–III)

In women with physiological pregnancy, no statistically significant changes in serum magnesium levels were observed between the first and third trimesters.

• Group I (magnesium deficiency): magnesium levels remained stable (*p* = 0.927; Cohen’s *d* < 0.2), indicating no meaningful intraindividual variation.
• Group II (magnesium insufficiency): no significant change was detected (*p* = 0.24; Cohen’s *d* < 0.2), with minimal variability (95% CI of change: −0.0217 to 0.0057 mmol/L).
• Group III (normal magnesium): a slight decrease was observed, but it was not statistically significant (*p* = 0.16; Cohen’s *d* < 0.2).

Overall, in physiological pregnancy, serum magnesium levels remained relatively stable regardless of baseline status. However, women with initial deficiency or insufficiency did not reach optimal reference values even after observation and/or supplementation.

### 3.3. Complicated Pregnancy (Groups IV–VI)

In women with complicated pregnancies, different patterns of magnesium dynamics were observed.

• Group IV (deficiency): no statistically significant change between trimesters was observed (*p* = 0.19; Cohen’s *d* = 0.27), although a slight upward trend was noted.
• Group V (insufficiency): a borderline decrease in magnesium levels was observed, approaching statistical significance (*p* = 0.054; Cohen’s *d* = −0.29).
• Group VI (normal magnesium): a statistically significant decrease in serum magnesium was detected in the third trimester (*p* = 0.043), with a moderate effect size (Cohen’s *d* = 0.41).

Overall, in complicated pregnancies, a tendency toward magnesium decline or instability was observed, particularly in women with initially normal magnesium levels.

### 3.4. Between‐Group Comparisons and Effect of Magnesium Therapy

**FIGURE 1:**
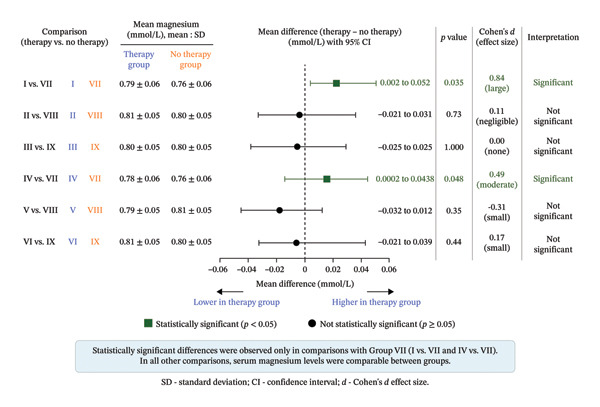
Intergroup comparison of serum magnesium levels between treated and untreated subgroups during pregnancy.

Serum magnesium levels were further compared between women receiving magnesium supplementation and those who did not (Groups VII–IX). Results are presented in Figure 1.

Significant differences were limited and mainly observed in comparisons involving the untreated subgroup (Group VII).

• Group I vs. Group VII: significantly lower magnesium levels were observed in the nontreated group (*p* = 0.035), with a large effect size (Cohen’s *d* = 0.84) and a 95% CI of mean difference of 0.002–0.052 mmol/L.
• Group IV vs. Group VII: a statistically significant difference was also observed (*p* = 0.048), with a moderate effect size (Cohen’s *d* = 0.49) and a narrow CI close to zero (0.0002–0.0438 mmol/L).

In contrast, no statistically significant differences were found in the remaining comparisons:

• Group II vs. Group VIII: *p* = 0.73; Cohen’s *d* = 0.11 (negligible effect).
• Group V vs. Group VIII: *p* = 0.35; Cohen’s *d* = −0.31 (small effect).
• Group III vs. Group IX: *p* = 1.000; complete overlap of 95% CI.
• Group VI vs. Group IX: *p* = 0.44; Cohen’s *d* = 0.17 (small effect).

### 3.5. Summary of Intergroup Findings

Overall, statistically significant differences between treated and untreated groups were limited and primarily observed in comparisons involving Group VII (untreated deficiency subgroup). In most other comparisons, serum magnesium levels remained comparable regardless of supplementation status.

These findings suggest that magnesium levels are relatively stable during pregnancy but may show subtle declines in complicated gestations and limited responsiveness in certain subgroups under standard oral correction therapy.

## 4. Discussion

In the present study, serum magnesium levels remained stable throughout pregnancy in women with physiological gestation, with no statistically significant changes observed between the first and third trimesters. In contrast, in women with complicated pregnancy, a tendency toward decreased serum magnesium levels was identified. This decline reached statistical significance in Group VI (*p* = 0.043) and approached significance in Group V (*p* = 0.054), indicating a progressive disturbance of magnesium homeostasis in pathological gestational conditions.

These findings are in agreement with previously published data demonstrating that serum magnesium concentrations tend to decrease with advancing gestational age, although the magnitude and statistical significance of this change may vary across studies. One report has shown that magnesium levels are lower in pregnant women compared to nonpregnant controls, and although differences between trimesters were not always statistically significant, a gradual decline with increasing gestational age was observed [19].

The observed alterations in magnesium status may be explained by its key physiological role in pregnancy. Magnesium is essential in the regulation of inflammatory pathways and oxidative stress balance. Deficiency of magnesium has been associated with enhanced inflammatory activity and impaired antioxidant defense mechanisms, both of which are implicated in the pathogenesis of pregnancy complications, including preterm labor [20]. Moreover, magnesium exerts anti‐inflammatory effects by modulating cytokine production and inhibiting inflammatory mediators [21].

Oxidative stress represents another important mechanism linking magnesium deficiency to adverse pregnancy outcomes. It is characterized by an imbalance between reactive oxygen species production and antioxidant defense systems. Magnesium contributes to the maintenance of cellular redox homeostasis and acts as a cofactor for multiple antioxidant enzymes, thereby protecting placental and maternal tissues from oxidative damage [22].

In addition, magnesium plays a fundamental role in the regulation of uterine contractility. It modulates calcium influx at the cellular level and thereby influences smooth muscle excitability. Proper uterine quiescence during pregnancy depends on a finely regulated balance between intracellular magnesium and calcium concentrations [23]. Disruption of this balance may increase uterine contractility and contribute to the initiation of preterm labor.

The interaction between magnesium and calcium signaling is also critical for hormonal regulation during pregnancy, particularly in relation to oxytocin‐mediated uterine contractions. Magnesium acts as a functional calcium antagonist, and its deficiency may enhance calcium‐dependent pathways, leading to increased uterine sensitivity to contractile stimuli [24, 25].

Clinical evidence further supports the association between low serum magnesium levels and adverse pregnancy outcomes. Significantly reduced magnesium concentrations have been reported in women who experience preterm birth, and these associations appear to be independent of maternal age, parity, gestational age, and socioeconomic factors. These findings suggest that serum magnesium may serve as a potential predictive biomarker for preterm delivery, with reported sensitivity and specificity reaching 98.0% and 88.0%, respectively [26, 27].

Similarly, low serum magnesium levels have been associated with hypertensive disorders of pregnancy, including preeclampsia. Studies have demonstrated that magnesium deficiency may precede the clinical onset of preeclampsia, suggesting a potential etiological role rather than a secondary consequence of the disease [28]. Proposed mechanisms include increased vascular tone due to calcium dominance and enhanced platelet activating factor (PAF) activity in the presence of magnesium deficiency, both of which contribute to endothelial dysfunction and hypertensive pathology [29].

Furthermore, logistic regression analyses have confirmed that low magnesium levels are independently associated with an increased risk of preeclampsia (OR: 0.25; 95% CI: 0.07–0.94; *p* = 0.04), reinforcing the potential predictive value of magnesium status in early pregnancy [30]. These findings are consistent with the results of the present study, which also demonstrated a relationship between reduced serum magnesium levels and the development of pregnancy complications.

Despite these associations, the clinical effectiveness of routine magnesium supplementation remains controversial. Several randomized controlled trials and meta‐analyses have not demonstrated a consistent reduction in obstetric complications following oral magnesium supplementation. Nevertheless, some evidence suggests that magnesium assessment during pregnancy may have predictive value for preterm birth risk stratification, and that supplementation may provide partial benefit in selected populations [31, 32].

A notable finding in the present study was the absence of full normalization of serum magnesium levels in women with baseline deficiency despite receiving oral magnesium therapy. This observation is consistent with literature reporting variable bioavailability of different magnesium formulations and limited efficacy of standard supplementation regimens in restoring serum concentrations to optimal levels [33].

These findings highlight the need for further research to clarify dose–response relationships, tissue bioavailability, and long‐term clinical effects of magnesium supplementation. In particular, future studies should evaluate whether magnesium supplementation has a preventive, therapeutic, or merely adjunctive role in high‐risk pregnancies [34].

Taken together, the results of this study suggest that:

• Serum magnesium demonstrates relative stability during physiological pregnancy.
• Magnesium alterations are more pronounced in complicated gestation.
• Serum magnesium alone may have limited diagnostic value as an isolated biomarker of total body magnesium status.
• Standard oral magnesium supplementation does not always ensure normalization of serum levels.

### 4.1. Limitations

Several limitations should be acknowledged. First, the observational design and absence of randomization limit the ability to establish causal relationships. Second, serum magnesium reflects only a small fraction of total body magnesium and does not necessarily represent intracellular magnesium status. Third, adherence to magnesium therapy was not objectively measured, which may have influenced treatment outcomes. Finally, some subgroup analyses were limited by relatively small sample sizes, potentially reducing statistical power.

## 5. Conclusion

In conclusion, the present study demonstrates stability of serum magnesium levels during physiological pregnancy and significant alterations in complicated gestation. The findings indicate that standard oral magnesium supplementation does not consistently normalize serum magnesium levels. These results support the hypothesis that serum magnesium has limited standalone diagnostic value and highlight the need for more sensitive biomarkers and individualized therapeutic strategies for magnesium deficiency during pregnancy.

## Author Contributions

Conceptualization, data collection, data analysis, statistical analysis, writing–original draft, and writing–review and editing: Hasmik Badalyan, Araksya Grigoryan, and Georgi Okoev.

## Funding

The authors declare that this study did not receive any specific funding.

## Disclosure

All authors have read and approved the final version of the manuscript. The corresponding author confirms that this manuscript is an accurate, transparent, and complete representation of the conducted research. No relevant data or methodological aspects have been omitted. Any deviations from the original study protocol (if applicable) have been clearly explained within the manuscript.

## Ethics Statement

The study was conducted in accordance with the Declaration of Helsinki. Ethical approval was obtained from the local institutional ethics committee. All participants provided written informed consent prior to inclusion in the study.

## Consent

Please see the Ethics Statement. All participants provided informed consent for publication of anonymized data.

## Conflicts of Interest

The authors declare no conflicts of interest.

## Data Availability

The data supporting the findings of this study are included within the article. Additional datasets are available from the corresponding author upon reasonable request.
